# The Relationships between Metabolic Disorders (Hypertension, Dyslipidemia, and Impaired Glucose Tolerance) and Computed Tomography-Based Indices of Hepatic Steatosis or Visceral Fat Accumulation in Middle-Aged Japanese Men

**DOI:** 10.1371/journal.pone.0149689

**Published:** 2016-03-03

**Authors:** Kazutoshi Fujibayashi, Toshiaki Gunji, Hirohide Yokokawa, Toshio Naito, Noriko Sasabe, Mitsue Okumura, Kimiko Iijima, Katsuhiko Shibuya, Teruhiko Hisaoka, Hiroshi Fukuda

**Affiliations:** 1 Department of General Medicine, School of Medicine, Juntendo University, Tokyo, Japan; 2 Center for Preventive Medicine, NTT Medical Center Tokyo, Tokyo, Japan; National Institute for Viral Disease Control and Prevention, CDC, China, CHINA

## Abstract

**Background:**

Most studies on the relationships between metabolic disorders (hypertension, dyslipidemia, and impaired glucose tolerance) and hepatic steatosis (HS) or visceral fat accumulation (VFA) have been cross-sectional, and thus, these relationships remain unclear. We conducted a retrospective cohort study to clarify the relationships between components of metabolic disorders and HS/VFA.

**Methods:**

The participants were 615 middle-aged men who were free from serious liver disorders, diabetes, and HS/VFA and underwent multiple general health check-ups at our institution between 2009 and 2013. The data from the initial and final check-ups were used. HS and VFA were assessed by computed tomography. HS was defined as a liver to spleen attenuation ratio of ≤1.0. VFA was defined as a visceral fat cross-sectional area of ≥100 cm^2^ at the level of the navel. Metabolic disorders were defined using Japan’s metabolic syndrome diagnostic criteria. The participants were divided into four groups based on the presence (+) or absence (-) of HS/VFA. The onset rates of each metabolic disorder were compared among the four groups.

**Results:**

Among the participants, 521, 55, 24, and 15 were classified as HS(-)/VFA(-), HS(-)/VFA(+), HS(+)/VFA(-), and HS(+)/VFA(+), respectively, at the end of the study. Impaired glucose tolerance was more common among the participants that exhibited HS or VFA (p = 0.05). On the other hand, dyslipidemia was more common among the participants that displayed VFA (p = 0.01).

**Conclusions:**

It is likely that VFA is associated with impaired glucose tolerance and dyslipidemia, while HS might be associated with impaired glucose tolerance. Unfortunately, our study failed to detect associations between HS/VFA and metabolic disorders due to the low number of subjects that exhibited fat accumulation. Although our observational study had major limitations, we consider that it obtained some interesting results. HS and VFA might affect different metabolic disorders. Further large-scale longitudinal studies are needed to reveal the relationships between the components of metabolic disorders and HS/VFA.

## Introduction

In recent years, obesity, elevated blood pressure, impaired glucose tolerance, and abnormal lipid metabolism, all of which are risk factors for arteriosclerosis-associated cardiovascular disease, have been grouped together into a disorder called “metabolic syndrome” [1,2]. Rather than all appearing at once, obesity, diabetes, hypertension, and dyslipidemia can occur at different times. Obesity, particularly the accumulation of abdominal fat, is considered to be the pathological basis of all of the other metabolic disorders. Abdominal fat accumulation is linked to insulin resistance, and insulin resistance and elevated insulin levels are known to cause hyperglycemia, hypertension, and dyslipidemia, which can lead to the development of arteriosclerosis-related coronary artery disease [1–4]. Accordingly, waist circumference, an indicator of the degree of abdominal visceral fat accumulation (VFA), is used as a diagnostic criterion for metabolic syndrome [5].

Currently, hepatic steatosis (HS), and non-alcoholic fatty liver disease (NAFLD) in particular, is widely considered to be a manifestation of metabolic syndrome. VFA and HS are related to increased gluconeogenesis, higher free fatty acid levels, and insulin resistance [6]. In addition, a strong correlation has been detected between HS and VFA [7]. That said, there are variants of HS that occur independently of VFA. Moreover, HS and VFA are independent risk factors for metabolic disorders [8,9]. Some studies have also suggested that they might be related to cardiovascular disease [10,11].

On the other hand, a longitudinal study suggested that HS is not an independent predictor of coronary events [12]. Most previous studies examining the associations between metabolic disorders and HS or VFA have been cross-sectional in nature, and thus, the causal relationships between HS or VFA and dyslipidemia, impaired glucose tolerance, hypertension, and other metabolic disorders remain unclear.

Abdominal ultrasonography and the measurement of waist circumference are accepted as useful initial screening tools for detecting fatty liver and VFA. Nevertheless, the sensitivity and specificity of these methods for detecting HS or VFA are a constant matter of debate. In addition, there are problems with the reproducibility of abdominal ultrasonography and waist circumference measurements. Although proton magnetic resonance spectroscopy can accurately assess liver fat content, it is difficult to examine large numbers of subjects using this method. In addition, many institutions do not have magnetic resonance imaging scanners. In previous studies, HS has often been evaluated by calculating the liver to spleen attenuation ratio (LSR) based on computed tomography (CT) data. CT exhibits a good degree of reproducibility and can be used to evaluate VFA and HS in large numbers of subjects. To the best of our knowledge, this is the first longitudinal observational study that has attempted to clarify the relationships between HS or VFA (according to CT-based evaluations of the LSR and the visceral fat cross-sectional area (VFT) at the level of the navel) and metabolic disorders or insulin resistance.

## Material and Methods

### Ethics Statement

The subjects’ clinical data were retrospectively retrieved from an institutional database. Before each examination, the participants were informed that the clinical data obtained during the program might be retrospectively analyzed and published. All of the examinations included in this study were performed as a routine part of the program, and none of them were specifically performed to collect data for the current study. Written informed consent was thus not required. On the other hand, participant records/information was anonymized and de-identified prior to analysis. And besides, access to the records/information has been strictly restricted to medical employers of NTT Medical Center Tokyo permitted by the institutional ethics committee. The study protocol was approved by the institutional ethics committee. The name of the Ethics Committee is "Ethics Committee of NTT Medical Center, Tokyo". The approval number is below; 13–26, 15–98.

### Study population

This was a retrospective cohort study conducted at the Center for Preventive Medicine at the Nippon Telegraph and Telephone Corporation (NTT) Medical Center, Tokyo, from April 2009 to March 2013. Employers in Japan are required by the Industrial Health and Safety Law to commission medical examinations once a year to ensure the health of their employees. We were contracted by NTT, a telecommunications company, to provide medical examinations to their employees based on the requirements of the abovementioned law. The present study was conducted as part of the general health check-up program delivered at our center. Most of the study subjects were employees of NTT or relatives of NTT employees. Our investigation focused on men who underwent more than two examinations at our center during the abovementioned period. For participants who underwent several examinations during the study period, their initial and latest data were analyzed. The exclusion criteria were as follows: subjects with severe diabetes that could not undergo the oral glucose tolerance test (OGTT), subjects with a history of liver disease (type B/C hepatitis, cirrhosis), subjects in which hepatitis B surface antigen/hepatitis C virus antibody was detected, and subjects for whom insufficient data were available. Based on the abovementioned exclusion criteria, a total of 1,106 subjects were enrolled in this study. However, 491 subjects who exhibited VFA or HS at the initial examination were also excluded. Thus, the data of 615 subjects were analyzed.

### Data collection

All examinations were performed at a single institution, and the results were used for the abovementioned mandated medical employee examinations. Therefore, the precision of the examinations did not vary. The participants completed self-administered questionnaires regarding their demographic characteristics and medical history, and well-trained staff interviewed any participants who had failed to complete the forms. The examinations were performed on two consecutive days. On the first day, weight and height were measured after the subject had removed their shoes and heavy clothing, and blood pressure was measured in the sitting position with an automatic monitor. In addition, serum samples were collected from each participant after overnight fasting and immediately subjected to biochemical analysis. The serum samples were also used to determine the subjects’ hemoglobin A1c (HbA1c), triglyceride, and high-density lipoprotein cholesterol (HDLC) levels. VFA was evaluated on CT scans obtained at the level of the navel; i.e., the VFT was calculated using the software program Fat Scan (East Japan Institute of Technology Co. Ltd., Ibaraki, Japan). The unenhanced CT protocol used in this study included a tube voltage of 120 kVp, a tube current of 60–75 mA, and slice thickness 5 mm. HS was evaluated using the LSR, which was calculated by dividing the mean hepatic CT attenuation value (Hounsfield units: HU) by the mean splenic CT attenuation value. The mean hepatic and splenic CT attenuation values were obtained using a validated single-slice unenhanced low-dose CT protocol for lung cancer and VFA screening. Specifically, they were obtained by calculating the mean CT attenuation values of two circular regions of interest (ROI) placed in each organ. For each ROI, we selected an appropriate region and avoided areas containing visible vascular or biliary structures in order to obtain accurate attenuation values for parenchymal tissue. On the second day, participants without obvious diabetes underwent 75-g OGTT examinations, in which we measured their fasting plasma glucose (FPG) levels, 1-hour and 2-hour post-loading plasma glucose concentrations, and immunoreactive insulin (IRI) levels.

### Data handling

The subjects’ HbA1c (Japan Diabetes Society; JDS) values were converted to National Glycohemoglobin Standardization Program (NGSP) values using the formula developed by the JDS (see below) [13,14].

The following parameters were calculated in this study:

(1) HbA1c = [HbA1c (JDS)(%)×1.02+0.25(%)]
(2) Simple Insulin Sensitivity Composite Index (ISI)[15,16] = [10,000/sqrt {FPG level (mg/dl)×fasting IRI level (μU/ml)×2-hour glucose level (mg/dl)×2-hour IRI level (μU/ml)}]

VFA was defined as a visceral fat cross-sectional area of ≥100 cm^2^ at the level of the navel [17]. Liver fat accumulation was defined as an LSR of ≤1.0 according to the findings of a previous report and our research [18]. Insulin resistance was assessed using the ISI. Metabolic disorders were defined using the following modified versions of the Japanese metabolic syndrome criteria [17]: high blood pressure: a systolic blood pressure level of ≥130 mmHg, a diastolic blood pressure level of ≥85 mmHg, or receiving treatment for hypertension; impaired glucose tolerance: an HbA1c value of ≥6.0%, an FPG level of ≥110 mg/dl, a 2-hour plasma glucose level during a 75-g OGTT of ≥140 mg/dl, or receiving treatment for impaired glucose tolerance; dyslipidemia: a triglyceride level of ≥150 mg/dl, an HDLC level of <40 mg/dl, or receiving treatment for dyslipidemia. The subjects were considered to have developed the abovementioned metabolic disorders when they fulfilled the relevant criteria. In addition, the incidence of metabolic disorders was calculated by dividing the number of subjects that newly developed metabolic disorders in each group by the total number of subjects in each group as shown below:

(3) The incidence of metabolic disorders (%) = the number of subjects in each group that had newly developed metabolic disorders at the latest examination / the total number of subjects (including subjects with metabolic disorders at the initial examination) in each group × 100.

Worsening insulin resistance was defined as a ΔISI *(latest ISI—initial ISI)* of less than 0. Elevated high sensitivity C-reactive protein (hsCRP) levels were defined as a ΔhsCRP *(latest hsCRP level—initial hsCRP level)* of more than 0. Body weight change was calculated by subtracting the subject’s BMI at the initial examination from their BMI at the latest examination. Exercising was defined as exercising twice a week or more based on the method employed a previous report [19]. Subjects who met this criterion at both examinations or at the time of the latest examination were included in the exercising group. Heavy alcohol consumption was defined as consuming alcohol five times a week or more according to the method described in a previous study [20]. Subjects who met the criterion for heavy alcohol consumption at both examinations or at the time of the latest examination were included in the heavy alcohol consumption group.

### Statistical analysis

Subjects who had LSR of ≤1.0 or VFT values of ≥100 cm^2^ at the initial examination were excluded as they were considered to exhibit HS or VFA, respectively. Based on the data obtained during the latest examination, the subjects were divided into four groups using an LSR cut-off level of 1.0 and a VFT cut-off level of 100 cm^2^; i.e., subjects who exhibited an LSR of >1.0 and a VFT of <100 cm^2^ were included in the HS(-)/VFA(-) group; subjects that displayed an LSR of >1.0 and a VFT of ≥100 cm^2^ were included in the HS(-)/VFA(+) group; subjects that demonstrated an LSR of ≤1.0 and a VFT of <100 cm^2^ were included in the HS(+)/VFA(-) group; and subjects that exhibited an LSR of ≤1.0 and a VFT of ≥100 cm^2^ were included in the HS(+)/VFA(+) group. The demographic characteristics of the subjects were compared among the groups using the m×n Chi-squared test or the Kruskal-Wallis test. Then, the associations between each group and the observed changes in insulin resistance, the hsCRP level, or body weight; the incidence of metabolic disorders; and lifestyle factors were investigated using the m × n Chi-squared test. All calculations were performed using the statistical software program JMP, version 9.0.2 (SAS Institute Inc., Cary, NC, USA). Continuous data are reported as median values (minimum value, maximum value). P-values of <0.05 were considered to be statistically significant.

## Results

Table 1 shows the characteristics of the study subjects at the initial examination.

**Table 1:** Demographic characteristics according to Hepatic steatosis and Visceral fat accumulation.

						*P value*
		*Median(minimum*, *maximum) or N (%)*				
	Overall	HS(-)/VFA(-)	HS(-)/VFA(+)	HS(+)/VFA(-)	HS(+)/VFA(+)	
Numbers of participants	615(100)	521(85)	55(9)	24(4)	15(2)	-
Examination interval (days)	757(196,1282)	749(196,1269)	763(199,1282)	774(261,1244)	1070(365,1175)	0.14
Age (years)	50(31,77)	50(31,77)	52(41,64)	45(40,64)	48(42,70)	0.11
Body mass index (kg/m^2^)	22.3(15.5,29.7)	22(15.5,27.7)	23.5(20,28.2)	23.6(19.5,29.7)	23.3(21.3,27.9)	<0.01
Visceral fat tissuue (VFT) (cm^2^)	61.5(11.5,99.9)	56.7(11.5, 99.5)	87.1(59.6, 99.7)	71.65(23,95.7)	90.2(69.5,99.9)	<0.01
Liver / Spleen Ratio (L/S Rtaio)	1.18(1.01,1.46)	1.19(1.01,1.46)	1.17(1.02,1.41)	1.10(1.01, 1.32)	1.11(1.02,1.23)	<0.01
High sensitive C reactive protein (hsCRP)	0.025(0.002,1.540)	0.022(0.002,1.540)	0.035(0.007,0.585)	0.0275(0.010, 1.040)	0.040(0.015,0.179)	<0.01
Glucose metabolism-related measurements						
Hemoglobin A1c (%)	5.6(4.5, 6.7)	5.6(4.5, 6.7)	5.6(4.8, 6.2)	5.6(4.7, 6.2)	5.6(5.0, 6.2)	0.67
75g oral glucose tolerance test						
Plasma glucose						
Fasting plasma glucose (mg/dl)	97(74,128)	97(74, 128)	98(84, 116)	95.5(82, 117)	99(91, 117)	0.25
1 hour—plasma glucose (mg/dl)	138(55,311)	134(55, 271)	161(67, 231)	163(101, 311)	161(84, 235)	0.01
2 hour—plasma glucose (mg/dl)	106(50,255)	104(50, 245)	120(72, 184)	114(66, 255)	115(94, 196)	<0.01
1 hour +2 hour plasma glucose (mg/dl)	240(134, 566)	236(134, 483)	272(165, 404)	279(175, 566)	273(196, 431)	<0.01
Sum of plasma glucose during OGTT(mg/dl)	338(220,683)	334(220, 603)	368(260, 498)	382.5(263, 683)	364(292, 534)	<0.01
Immunoreactive insulin (IRI)						
Fasting IRI (μU/ml)	5.0(1.0,14.8)	4.8(1.0,14.8)	5.4(2.5, 13.7)	6.6(2.7,13.4)	5.6(2.7,11.7)	<0.01
1 hour—IRI (μU/ml)	42.9(2.8,197.6)	41.5(2.8, 197.6)	49.8(10.2, 151.9)	51.15(23.6, 149.4)	48.3(11.4, 116.1)	<0.01
2 hour—IRI (μU/ml)	29.1(3.2,184)	27.5(3.2, 184.0)	39.2(7.6, 180.9)	31.1(16.5, 127.3)	47.6(35.4, 90.4)	<0.01
1 hour + 2 hour—IRI (μU/ml)	72.3(15.7, 332.5)	69.8(15.7, 332.5)	90.6(22.2, 293.5)	96.9(61.3, 272.2)	94.0(49.5, 200.6)	<0.01
Sum of IRI during OGTT(μU/ml)	78.5(20.6,343.1)	74.9(20.6, 343.1)	99.4(25.8, 307.2)	101.85(64.3, 283.9)	100.2(55.1, 206.1)	<0.01
Insulin metabolism parameters						
Simple insulin sensitivity composite index (ISI)	8.3(1.8,52.4)	8.9(1.9, 52.4)	6.6(1.8, 22.4)	6.9(2.3, 13.7)	5.8(2.8, 7.3)	<0.01
Subjects with impaired glucose tolerance (%)	142(23.1)	113(21.7)	17(30.9)	8(33.3)	4(26.7)	0.29*
Blood pressure measurements (mmHg)						
Systolic	124(88,173)	124(88, 173)	128(96, 171)	122.5(111, 160)	129(110,159)	0.12
Diastolic	80(57,117)	79(57, 117)	83(62, 107)	78(68, 102)	80(69, 104)	0.04
Subjects with high blood pressure (%)	249(40.5)	206(39.5)	28(50.9)	6(25.0)	9(60.0)	0.06*
Lipid-metabolic related measurements						
High density lipoprotein—cholesterol (mg/dl)	60(28,123)	61(28,123)	59(33,106)	54.5(39,113)	48(40,96)	0.03
Low density lipoprotein—cholesterol (mg/dl)	113(32,191)	113(32,183)	114(64,191)	110(66, 161)	122(95,186)	0.43
Triglyceride (mg/dl)	86(23,469)	83(23,469)	107(40,322)	103.5(26,237)	129(48,353)	<0.01
Subjects with dyslipidemia (%)	116(18.9)	83(15.9)	17(30.9)	10(41.7)	6(40.0)	<0.01*

the Kruskal-Wallis test was used;

* the m × n Chi—squared test was used

Hepatic Steatosis was abbreviated as HS, Visceral Fat Accumulation was abbreviated as VFA

The median examination interval was 757 days, and the median age of the subjects was 50 years. The subjects were classified into four categories according to the presence/absence of HS and VFA at the latest examination as follows: HS(-)/VFA(-), 521 subjects; HS(-)/VFA(+), 55 subjects; HS(+)/VFA(-), 24 subjects; and HS(+)/VFA(+), 15 subjects. In other words, HS or VFA was observed in 94 (15%) subjects. At the initial examination, the HS(+)/VFA(-) subjects displayed the highest plasma glucose level sums during the OGTT, the highest post-loading IRI levels, and the highest prevalence of impaired glucose tolerance. On the other hand, the HS(+)/VFA(+) subjects demonstrated the worst insulin resistance and HDLC/triglyceride levels. With respect to blood pressure measurement, both the HS(-)/VFA(+) subjects and the HS(+)/VFA(+) subjects may display higher blood pressure levels and a higher prevalence of high blood pressure than the other groups. Table 2 shows the associations between HS/VFA and the examined metabolic disorders.

**Table 2:** The relationships between hepatic steatosis / visceral fat accumulation and metabolic disorders.

	*N (%)*				*P value*
	HS(-)/VFA(-)	HS(-)/VFA(+)	HS(+)/VFA(-)	HS(+)/VFA(+)	
incidence of worsening insulin sensitivity	214(41)	27(49)	13(54)	10(67)	0.10
incidence of worsening high sensitive C reactive protein	205(39)	28(51)	11(46)	12(80)	<0.01
incidence of high blood pressure	46(9)	9(16)	4(17)	2(13)	0.25
incidence of impaired glucose tolerance	32(6)	7(13)	4(17)	3(20)	0.05
incidence of dyslipidemia	41(8)	13(24)	1(4)	2(13)	<0.01

the m × n Chi—squared test was used; Hepatic Steatosis was abbreviated as HS, Visceral Fat Accumulation was abbreviated as VFA

The incidence of impaired glucose tolerance seemed to differ between the groups (p = 0.05). The subjects that exhibited VFA or HS appeared to display a high incidence of impaired glucose tolerance. In addition, the incidence of impaired glucose tolerance seemed to be markedly higher among the subjects with HS than among those without HS. On the other hand, the incidence of dyslipidemia increased in association with VFA (p<0.01). Furthermore, the greatest increases in the hsCRP level were observed in the subjects with both HS and VFA (p<0.01). Lastly, insulin resistance seemed to deteriorate in the patients with both HS and VFA than in those that only exhibited VFA (p = 0.11).

Table 3 shows the associations between HS/VFA and body mass index (BMI) changes/lifestyle factors.

**Table 3:** The associations between hepatic steatosis / visceral fat accumulation and body mass index change / life style.

	*N (%)*				
	HS(-)/VFA(-)	HS(-)/VFA(+)	HS(+)/VFA(-)	HS(+)/VFA(+)	*P value*
0≥ΔBMI	268(52)	9(16)	8(33)	4(27)	
0<ΔBMI≤0.6	158(30)	18(33)	9(38)	2(13)	
0.6<ΔBMI	95(18)	28(51)	7(29)	9(60)	<0.01
Exercise habits	209(40)	16(29)	4(17)	4(27)	0.03
Heavy alcohol consumption	228(44)	29(53)	8(33)	4(27)	0.19

the m × n Chi—squared test was used; Hepatic Steatosis was abbreviated as HS, Visceral Fat Accumulation was abbreviated as VFA, Body Mass Index was abbreviated as BMI

Body weight increases of >0.6 BMI points were more common among the subjects that demonstrated VFA. On the other hand, the relationship between the body weight increases and HS was unclear. With regard to lifestyle factors, the subjects with HS alone exercised less often (p = 0.03). We did not detect a clear relationship between fat accumulation and alcohol consumption.

## Discussion

Unfortunately, contrary to our expectations, only a few subjects exhibited fat accumulation. Our observational study had several major limitations. First, the low number of subjects that demonstrated fat accumulation makes it difficult to generalize the results of this observational study. Second, the low number of subjects exhibiting fat accumulation means that it was not possible to adjust for biases or confounding factors using statistical methods. Thus, our study was not able to determine the individual effects of HS and VFA. Although our observational study suffered from the abovementioned major limitations, we consider that it obtained some interesting results.

Our longitudinal observational study suggested that HS and VFA are associated with different metabolic disorders. Specifically, HS seemed to be associated with impaired glucose tolerance, while VFA appeared to be associated with dyslipidemia and impaired glucose tolerance. In addition, insulin resistance seemed to deteriorate in patients with both HS and VFA than in those that only exhibited VFA.

In our study, about 26% (24/94 participants) of the patients in which some form of fat accumulation was observed exhibited HS, but not VFA. Severe insulin resistance and impaired glucose tolerance have already been shown to be important pathological factors in arteriosclerotic diseases. Thus, based on this fact and the results of the present study, it is likely that HS is a key pathological factor in arteriosclerotic diseases.

We obtained several other intriguing findings in this study: (1) the HS(+)/VFA(-) patients did not display clear patterns of weight gain, while the patients that exhibited VFA were often overweight; (2) the sum of the serum IRI levels recorded during the OGTT and the sum of the serum IRI levels at 60 min and 120 min after loading during the OGTT were both highest among the HS(+)/VFA(-) patients; and (3) the frequency of exercising was lowest among the HS(+)/VFA(-) patients. Many previous studies have shown that insulin resistance and elevated insulin levels may play an important role in NAFLD [21–24]. In addition, these conditions can appear in the absence of marked obesity [24,25]. Previous studies have suggested that physical inactivity is associated with fatty liver [26,27]; hepatic fat accumulation is associated with insulin resistance in skeletal muscle [28,29]; and insulin resistance in skeletal muscle may increase hepatic fat levels [30,31]. These findings and our observations regarding the HS(+)/VFA(-) patients indicate that a lack of exercise and elevated insulin levels might be risk factors for HS, even in the absence of weight gain or VFA. Currently, the general belief regarding metabolic disorders is that it begins with obesity or VFA and is followed by increased insulin resistance, which is associated with greater fat accumulation in the liver. However, it is possible that a vicious cycle occurs, with HS appearing before VFA and causing increased insulin resistance, which then triggers impaired glucose tolerance, and hence, promotes other metabolic disorders and arteriosclerotic diseases.

In the current study, the HS(+)/VFA(+) patients demonstrated the highest prevalence of elevated hsCRP levels, the most severe insulin resistance, and the highest prevalence rates of impaired glucose tolerance and dyslipidemia. Chronic inflammation is a key pathological factor in arteriosclerosis and is suggested to be related to cardiovascular events [32]. In addition, it has been reported that VFA is related to more severe fatty liver fibrosis and inflammation, which are characteristics of worsening NAFLD [33]. Based on the findings of the present study, HS and VFA might exacerbate each other. This suggests the importance of monitoring HS when managing arteriosclerotic diseases.

## Conclusion

The present longitudinal data analysis suggests that HS is a key pathological factor in metabolic disorders and/or insulin resistance. In addition, it is likely that HS and VFA are associated with different aspects of metabolic disorders. It is therefore important to consider HS. We consider that HS should be managed carefully in order to prevent adverse outcomes. However, our study was not able to determine the individual effects of HS and VFA. A further large-scale observational longitudinal study is necessary to determine these effects.

### Limitations

In addition to the abovementioned limitations, our study had several further limitations. First, it was affected by selection bias as it only involved males. In addition, more than 70% of the participants were healthy office workers who ranged in age from 40- to 60-years-old, and hence, the investigated cohort was affected by the healthy workers effect and did not accurately represent the Japanese population. Second, some of the parameters that were used in this study to evaluate liver fat accumulation, VFA, and insulin resistance are not considered to be gold standard methods. In particular, the LSR can not be used to assess total liver fat content, inflammation severity, or fibrosis. Therefore, we can not discuss the differences between non-alcoholic steatohepatitis and non-alcoholic fatty liver. Lastly, the subjects’ lifestyle habits and medical histories were evaluated using a self-administered questionnaire, and the subjects might have provided inaccurate information. Thus, it is possible that the subjects were not as healthy as our data suggested, and further evaluations of lifestyle habits/medical histories based on an established questionnaire are necessary.

## Supporting Information

S1 Data The data set that was analyzed for this study. (XLSX)
